# A Causal Role of Area hMST for Self-Motion Perception in Humans

**DOI:** 10.1093/texcom/tgaa042

**Published:** 2020-07-30

**Authors:** Constanze Schmitt, Bianca R Baltaretu, J Douglas Crawford, Frank Bremmer

**Affiliations:** 1 Department of Neurophysics, University of Marburg, Marburg, Germany; 2 Center for Mind, Brain and Behavior-CMBB, University of Marburg and Justus-Liebig-University Giessen, Germany; 3 International Research Training Group 1901: The Brain in Action; 4 Centre for Vision Research and Vision: Science to Applications (VISTA) Program, York University, Toronto, Ontario, Canada; 5 Department of Biology, York University, Toronto, Ontario, Canada; 6 Departments of Psychology, Biology, Kinesiology and Health Science, York University, Toronto, Ontario, Canada

**Keywords:** heading, medial-superior-temporal area, self-motion, transcranial magnetic stimulation, visually guided navigation

## Abstract

Previous studies in the macaque monkey have provided clear causal evidence for an involvement of the medial-superior-temporal area (MST) in the perception of self-motion. These studies also revealed an overrepresentation of contraversive heading. Human imaging studies have identified a functional equivalent (hMST) of macaque area MST. Yet, causal evidence of hMST in heading perception is lacking. We employed neuronavigated transcranial magnetic stimulation (TMS) to test for such a causal relationship. We expected TMS over hMST to induce increased perceptual variance (i.e., impaired precision), while leaving mean heading perception (accuracy) unaffected. We presented 8 human participants with an optic flow stimulus simulating forward self-motion across a ground plane in one of 3 directions. Participants indicated perceived heading. In 57% of the trials, TMS pulses were applied, temporally centered on self-motion onset. TMS stimulation site was either right-hemisphere hMST, identified by a functional magnetic resonance imaging (fMRI) localizer, or a control-area, just outside the fMRI localizer activation. As predicted, TMS over area hMST, but not over the control-area, increased response variance of perceived heading as compared with noTMS stimulation trials. As hypothesized, this effect was strongest for contraversive self-motion. These data provide a first causal evidence for a critical role of hMST in visually guided navigation.

## Introduction

Successful navigation through an environment is based on the integration of visual, vestibular, tactile, and auditory information (e.g., Hlavacka et al. 1996; Angelaki et al. 2011; von Hopffgarten and Bremmer 2011; Churan et al. 2017). Numerous behavioral studies in humans have shown that visual optic flow, which results from self-motion, alone can be used to determine the direction of one’s self-motion (heading) with high accuracy (e.g., Gibson 1950; Warren and Hannon 1988; Lappe et al. 1999; Lich and Bremme 2014).

Neurophysiological studies in the animal model of human sensorimotor processing, the macaque monkey, have revealed 2 cortical regions to be significantly involved in the encoding of visual self-motion information: the medial superior temporal area (area MST) (Saito et al. 1986; Duffy and Wurtz 1991; Lappe et al. 1996; Paolini et al. 2000; Britten and van Wezel 2002; Gu et al. 2006; Bremmer et al. 2010; Angelaki et al. 2011; Gu et al. 2012) and the ventral intraparietal area (area VIP) (Bremmer et al. 2002; Schlack et al. 2002; Chen et al. 2011; Kaminiarz et al. 2014; Shao et al. 2018). Based on these studies, it is generally assumed that heading perception is linked to the readout from population activity (e.g., Lappe et al. 1996; Bremmer et al. 2002; Ben Hamed et al. 2003; Britten 2008; Chen et al. 2018). While these studies have documented “correlations” between population activity and perception, results from experiments employing electrical microstimulation or reversible inactivation provided strong evidence for a “causal” role of area MST in monkeys’ heading perception (stimulation: Britten and van Wezel 1998; inactivation: Gu et al. 2012). The question, if also area VIP plays such a causal role is not fully resolved yet. While an earlier study provided evidence of such a view (Zhang and Britten 2011), a more recent study challenged it (Chen et al. 2016).

Neurons in both areas, MST and VIP, are tuned for visually simulated and real self-motion in full 3D space. Depending on what range of possible heading directions are provided in an experimental setting, mathematical functions, which quantify a neuron’s self-motion tuning, range from modified sinusoid function (MSF) for full 3D motion space (Gu et al. 2006), via sigmoidal functions for stimuli simulating forward motion with horizontal and vertical components (Lappe et al. 1996) to purely linear functions when simulating forward and slightly lateral motion across a horizontal plane (Bremmer et al. 2017). In addition to these findings, recent studies also demonstrated a significant overrepresentation of neurons encoding contraversive headings: there are more neurons in a right cortical hemisphere coding for leftward (contraversive) than for rightward (ipsiversive) self-motion (Kaminiarz et al. 2014; Greenlee et al. 2016), and vice versa.

Functional equivalents of both areas, MST and VIP, have been identified in humans (hMST: Dukelow et al. 2001; Huk et al. 2002; VIP: Bremmer et al. 2001) and their involvement in the processing of visual self-motion information has been documented (e.g., Morrone et al. 2000; Smith et al. 2006; Wall et al. 2008; Wall and Smith 2008; Amano et al. 2009; Pitzalis et al. 2013; Strong et al. 2017a). Like in the monkey, these studies found correlations between neural activity and certain features of visual self-motion stimuli. Clear evidence of a causal role for heading perception, however, is still lacking.

On-line transcranial magnetic stimulation (TMS) (delivered during behavior) has been shown to induce a phasic perturbation of neural activity, which might be interpreted as the injection of random noise relative to the behavior-related signal (e.g., Rossi et al. 2009; Prime et al. 2010; Thut and Pascual-Leone 2010; Taylor and Thut 2012; Valero-Cabré et al. 2017; Romero et al. 2019). Accordingly, this experimental tool is often used to test causal relationships between neural activity in certain brain regions and behavior (e.g., Vesia et al. 2010; Dessing et al. 2013). To our best knowledge, this has not been used to link hMST to the perception of heading.

Due to hMST’s accessibility to TMS, but not hVIP’s, and due to the unresolved question, if area VIP is causally involved in heading perception at all, here, we focused on probing the causal role of area hMST. We hypothesized that TMS should lead to a disturbance, that is, greater variance in the perception of heading, but not overall accuracy. Importantly and due to an overrepresentation of contraversive heading (Greenlee et al. 2016), this greater perceptual variance should be strongest for headings contraversive to the TMS stimulated hemisphere. We employed structural and functional magnetic resonance imaging (fMRI)-guided TMS to stimulate the human functional equivalent of macaque area MST (hMST) and a control area approximately 1.5 cm posterior to area hMST. We hypothesized impaired heading performance (i.e., reduced precision) due to TMS applied over hMST but not over the control area, nor for a no-stimulation condition.

## Materials and Methods

### Participants

Prior to our experiments, we performed a power analysis (G*Power, Faul et al. 2009) to determine the number of participants required for our study. We based our calculations on the effect size reported in a previous, most closely related study by Strong et al. (2019). Accordingly, 8 subjects participated in our study (6 females, 2 males, mean age: 22 years, ranging from 20 to 31). The participants had normal or corrected-to-normal vision. fMRI data were collected on a single day for each participant. TMS data were collected on 1, 2, or 4 days, depending on the individual availability of our participants. Participants were compensated with $25 CAD per hour for the fMRI experiment and $20 CAD per hour for the TMS experiment. Our study was in line with the Declaration of Helsinki and approved by York University Human Participants Review Committee within the context of the York Senate Policy on Research Ethics. Prior to each part of the experiment, participants provided written informed consent. The participants did not report any side effects attributed to the TMS besides the feeling of some discomfort during the stimulation.

### Functional Localizer and Control Site

We aimed to stimulate area hMST because of its potential role in processing self-motion information (Morrone et al. 2000; Wall et al. 2008; Wall and Smith 2008). Therefore, we aimed to determine the exact location of area hMST, as well as an appropriate control area on a subject-by-subject level. We analyzed the fMRI data in 2 steps. First, we aimed to identify areas sensitive to visual motion. These include area hMST, but also other areas like the human middle temporal area (hMT: Wall and Smith 2008) and the functional equivalent of macaque area VIP (Bremmer et al. 2001). In order to narrow down our region of interest to area hMST, we determined in a second step the part of the human MT/MST complex that responded not only to visual stimulation in the contralateral part of the visual field (hMT), but also to stimulation in the ipsilateral part of the visual field (hMST) (Dukelow et al. 2001; Smith et al. 2006).

#### MRI Setup

Once participants felt comfortable with the requirements of the localizer task, they were asked to lie flat on the MRI table. They were then fit with a 20-channel head coil. A head-mounted apparatus containing a mirror was placed above the coil in order to reflect images from the screen in the MRI bore, along with a head-mounted eye tracker (to track movements of the right eye). Head motion and eye movements were inspected offline.

#### Localizer Stimulus

**Figure 1:**
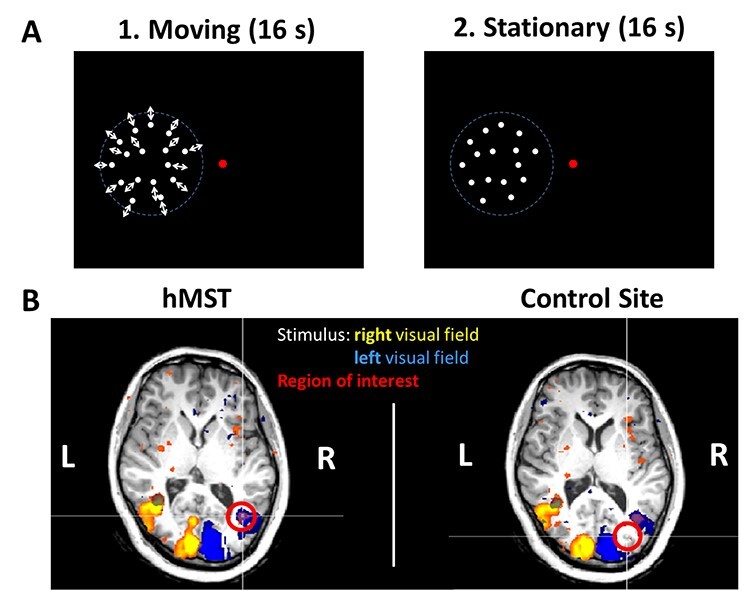
Functional localizer stimulus and results of one representative participant. In *A,* the stimulus for the functional localizer is plotted. It consisted of 2 phases. First, in a moving phase white dots were moving radially in- and outwards (1 s for each movement direction) for 16 s on a black background. In the second, the stationary phase, the dots were just displayed without movement for 16 s. There was always a fixation target displayed in the middle of the screen, which changed between 3 different gray levels, black and white. Here, we plotted only the case for visual stimulation left of the fixation target, but in a second block the same stimulus was presented with the dots right of the fixation target. In *B*, 2 axial plane slices of a functional scan of one participant are plotted. In the left slice, we marked area MST with a red circle and in the right slice the control site. Both TMS stimulation sites are in the right hemisphere. We colored the voxels which showed a significant difference between the 2 phases “moving” and “stationary.” In yellow data collected in sessions with stimulus presentation on the right half of the monitor and in blue data from stimulus presentation on the left side of the monitor are presented.

To identify our regions of interest (ROIs), we presented a stimulus based on previous studies to identify motion specific areas (Dukelow et al. 2001). The localizer stimulus consisted of a central fixation target and random white dots in a circular field with a radius of 8° either on the left or on the right of the fixation target on a black background (Fig. 1*A*). Every trial consisted first of a moving phase, followed by a stationary phase, each of which lasted 16 s.

In the moving phase, a 100% coherent expansion and contraction movement of the dots occurred every second. In the stationary phase, the dots were stationary within the circular aperture. We presented 8 trials per block and 2 blocks per participant. In one block, the dots were presented on the left side of the fixation target; in the other block, the dots were shown on the right side of the fixation target. For this task, participants had to fixate the central target: a bullseye with a white surrounding circle, a black inner circle, and a dot in the middle that changed contrast every 0.5 s. We added a counting task to keep participants attentive to the fixation target. The inner dot of the fixation target changed randomly between 3 different gray values, black and white throughout the experiment. Participants had to count how often the fixation target was white. This occurred between 5 and 15 times per block. After each block, they reported this number verbally.

#### Imaging

We collected imaging data at York University (Toronto, ON, Canada), using the 3.0 Tesla MRI scanner (Siemens Magnetom TIM Trio). We acquired functional data using an echo-planar imaging sequence (repetition time [TR] = 2000 ms; echo time [TE] = 30 ms; flip angle [FA] = 90°; field of view [FOV] = 240 mm, matrix size: 80 × 80 with an in-slice resolution of 3 × 3 mm; slice thickness = 3.5 mm, no gap). These parameters were the same for each of the 2 blocks per participant. Data were collected in an interleaved and ascending order. Thirty-three slices were obtained for each volume; 210 volumes were collected in total. In each experimental session, a T1-weighted anatomical reference volume was obtained using an MPRAGE sequence (TR = 2300 ms; FA = 9°; voxel size = 1 × 1 × 1 mm^3^). For each volume of anatomical data, 192 slices were collected.

#### Analysis

##### Preprocessing

In a first step, we screened the functional data for head movements that would produce artifacts. To this end, slice scan time correction (cubic spline), temporal filtering (to remove frequencies <2 cycles/run), and 3D motion correction (trilinear/sinc) were applied to the data (BrainVoyager QX 2.8, Brain Innovation). Volumes that showed (abrupt) movements greater than 2 mm were removed from our data set as confound predictors. Data that remained after preprocessing were then coregistered via gradient-based affine alignment (translation, rotation, and scale affine transformation) to the raw anatomical data. We, then, applied spatial smoothing using an FWHM of 8 mm to thedata.

##### fMRI Analysis

For each participant’s data, we used a general linear model (GLM) with 5 predictors. There was a predictor for each of our 4 conditions (“movement_right,” “stationary_right,” “movement_left,” and “stationary_left”). Each of these predictors had a duration of 16 s or 8 volumes. The fifth predictor was a baseline predictor at the beginning of each block. It lasted for 10 s or 5 volumes. A standard hemodynamic response function (HRF; Brain Voyager QX’s default double-gamma HRF) was convolved with the predictor variables using a rectangular wave function. GLMs were modified to ensure that confound predictors were included for trials in which there was excessive head motion. If more than 50% of the trials in a single run were modeled by a confound predictor, then that entire run was excluded from any further analysis.

#### TMS Control Site

Our selection of the control area was based on 2 boundary conditions. First, the control area must not have been activated by the visual stimulus. This argued for an area remote from the stimulation site. Second, based on findings reported by Dessing et al. (2013), the control area should be located close to the stimulation site. Their conclusion was drawn from the fact that subjects experience tactile sensations from TMS stimulation. If target and control areas are located sufficiently close to one another, the tactile sensations induced by the TMS pulses in the 2 stimulation conditions (target vs. control) do not differ and, hence, should not produce different effects on the TMS-induced modulation of visual processing. Based on these 2 boundary conditions, we decided to stimulate over a location approximately 1.5 cm posterior to the area we identified as hMST.

### Model of Heading Decoding

We aimed to predict the behavioral outcome of our study based on a recently introduced model of heading decoding (Bremmer et al. 2017). The quantitative model was built after neurophysiological data from the macaque monkey (Bremmer et al. 2010; Kaminiarz et al. 2014).

**Figure 2:**
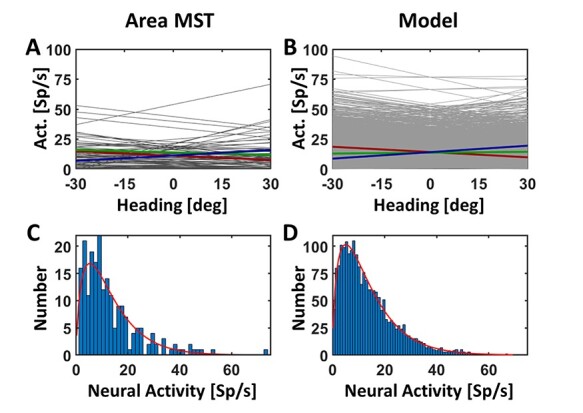
Activity distribution from experimental and model data. Panels *A* and *B* depict the distribution of regression lines shown in gray fitted to our real (*A*) or model (*B*) discharges. Average values for neurons tuned to leftward (contraversive) heading are shown in red, while average values for neurons tuned to rightward (ipsiversive) or straight-ahead self-motion are shown in blue (rightward) or green (straight-forward), respectively. Panels *C* and *D* depict the distribution of real (*C*) or model (*D*) responses to the 3 self-motion directions, classified into bins and fitted by gamma distributions (red lines).

**Figure 3:**
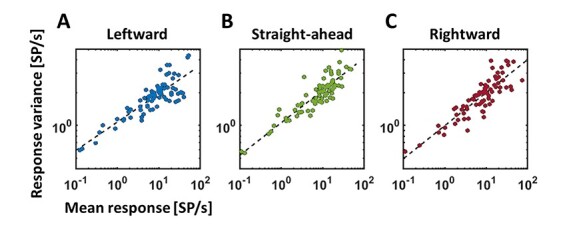
Response variance as a function of discharge. Responses from neurons in area MST had been recorded previously (Bremmer et al. 2010). From those neurons with a statistically significant response to simulated self-motion across a ground plane, we analyzed response variance for a given heading direction (results for leftward heading are displayed in A, for heading straight ahead in B and for rightward heading in C). This variance could be fitted with a power function *y* = a*x^b^, shown in a log-log format. Fit parameters for our model neurons were derived from responses to all 3 heading directions.

Most important features are (1) a linear dependency of neural activity on self-motion direction and (2) an overrepresentation of heading contraversive with respect to the recorded hemisphere. In order to make predictions concerning the effect of TMS on heading judgments, we extended our model. First, we analyzed activity profiles of neurons from area MST (Bremmer et al. 2010): we determined the distribution of (1) the slopes of the regression functions and (2) the discharges for the 3 self-motion directions at a single cell and at the population level (Fig. 2*A* and *C*). For the latter, we determined for each neuron with a significant response (*n* = 79) its average activity for each of the 3 self-motion directions. These 79 × 3 = 237 values were classified into bins, with a bin-width of 1.5 Sp/s. The resulting distribution of responses is shown as histogram in Figure 2*C* and was quantified by fitting a gamma distribution (Li et al. 2018). From these quantitative values, we created a sample of *n* = 2000 artificial neurons by generating 2000 random numbers, that is, discharges, from a gamma distribution with the shape parameter *a* and the scale parameter *b* derived from our experimental data (Fig. 2*D*) [MATLAB function: gamrnd()]. Figure 2*D* shows the resulting activity distribution for the 3 self-motion directions of these 2000 model neurons as a histogram, with activity being classified into bins with a bin-width of 0.75 Sp/s. This higher resolution, that is, half the bin-width, in Figure 2*D* as compared with Figure 2*C* was allowed by the larger number of values to be classified for the model neurons (3*2000 = 6000 values) as compared with the real neurons (237 values). Regression values were random numbers replicating the normal distribution observed in the recorded sample of MST neurons (Fig. 2*B*) [MATLAB function randn()]. Based on our own data (Greenlee et al. 2016), 45% (*n*_contra_ = 900) of our model neurons were tuned for contraversive (leftward) heading, 35% (*n*_ipsi_ = 700) for ipsiversive (rightward) heading and 20% (*n*_straight-ahead_) = 400 for straight-ahead self-motion. After creating this sample of neurons, we added noise to the neural activity to mimic the real discharges as closely as possible. Confirming numerous previous results, response jitter varied as a function of discharge and could be best quantified by a power function *y* = *ax*^b^, with *y* depicting the response jitter and *x* the mean response (Fig. 3). In a next step, we introduced the effect of TMS on neuronal activity. This effect of TMS was modeled as a disturbance of neural activity, as it can enhance or suppress neural activity (Hallett 2007; Valero-Cabré et al. 2017). Since we did not have a clear hypothesis concerning the absolute strength of TMS, we varied it between ±4% and ±40% of the response strength of each neuron, in steps of 4%. More specifically, for each neuron, we drew a value from a uniform distribution ranging from 96% to 104% of a neuron’s discharge to values ranging from 60% to 140% of a neuron’s discharge. This “artifical-TMS (aTMS)” was applied to all neurons alike, that is, neurons tuned for contraversive, ipsiversive, or straight-ahead self-motion.

**Figure 4:**
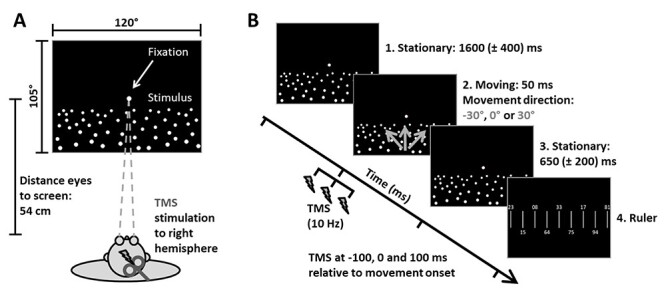
Behavioral/TMS setup and stimulus. In *A*, the setup of the behavioral/TMS experiment is presented. The participants were sitting in front of a 120° × 105° screen (distance eye to screen: 54 cm). The stimulus was presented on this screen while right hemisphere was stimulated with a TMS coil. In *B*, the stimulus is depicted. In a first stationary phase, a ground plane consisting of white random dots was displayed for 1600 (±400) ms on a black background. In the second, the moving phase, self-motion (gray arrows) to one of 3 headings (30° to the left, straight forward, 30° to the right) was simulated by a movement of the dots for 50 ms. After the movement, in a third phase, the ground plane was again presented stationary for 650 (±200) ms. Throughout these 3 phases, a fixation target was displayed in the center of the screen. In the fourth phase, a ruler stimulus was presented. It showed vertical lines which were 1° apart and random numbers alternating above and below each line. Participants were asked to type in the number corresponding to their perceived heading. In 57% of all trials, a train of 3 TMS pulses was delivered. The timing of the pulses was fixed: −100, 0, and 100 ms relative to movement onset.

In a final step, we decoded heading from the population of neurons. Here, the decoding-weight of a given neuron was proportional to its average firing rate (weighted decoding). This whole procedure was repeated 100 times. We compared the effect of aTMS by subtracting the mean decoded heading variance without aTMS from the value obtained under aTMS for each of the 3 heading directions. These difference values were tested for significant differences across headings with an analysis of variance (ANOVA) on ranks.

### Behavioral/TMS Experiment

#### Setup

Participants sat on a chair in a darkened room. We used individual bite bars for each participant in order to stabilize the head when participants were seated in front of the center of the screen (Heuer et al. 2016). Self-motion stimuli were back projected on the screen (185 cm wide and 140 cm high) that was 54 cm away from the participant’s eyes (Fig. 4*A*). The refresh rate of the projector was set to 60 Hz and the resolution to 1280 × 960 pixels. Participants used the number pad of a normal computer keyboard to provide their responses (for the exact task: see below).

An Eyelink 2 system (SR Research) was used to record and account for the participant’s eye movements. The Eyelink data were analyzed online and trials in which participants did not maintain fixation on the central target were aborted and repeated later in the experiment.

#### Neuronavigation

During the experiment, we constantly controlled the position of the TMS coil, so that we could stimulate the ROI as accurately as possible. We used the Brainsight TMS software (Brainsight TMS, Rogue Research Inc.).

Prior to a TMS session, we first loaded the anatomical fMRI scans which we had collected in the localizer stimulus session and determined the axis from the anterior to the posterior commissure (AC-PC). Next, the skin was reconstructed and we set landmarks (the tip of the nose, the nasion, and both preauricular sites) for the calibration of our tracking system. We then entered in the predefined locations for the areas we wanted to stimulate.

Before each TMS session, we calibrated the location of the TMS coil and the landmarks on the participant’s head using the Brainsight motion tracker system. Visual feedback was provided to the experimenter during the entire experiment, which showed the 3D distance between a vector through the center of the figure-of-eight coil and the ROI. This image could only be seen by the experimenter on a dimly lit monitor. The TMS coil was connected to an adjustable holding device to reduce the weight. During the experiment, the coil was positioned and held manually by the experimenter above the head of the participant to stimulate the ROI as accurately as possible.

#### Experimental Stimulus

In the behavioral and TMS part of our study, we presented a stimulus simulating self-motion across a ground plane made of white random dots in otherwise darkness. The location of the fixation target was on the vertical meridian, slightly above the horizon. Each trial consisted of 3 phases. The first lasted between 1200 and 2000 ms and showed the stationary ground plane. The immediately following second phase (50 ms) simulated a forward self-motion into one of 3 directions (Fig. 4*B*): 30° to the left, straight ahead, or 30° to the right. In the third phase, right after the movement, we presented a ruler with random numbers which was used to identify the perceived heading (Kaminiarz et al. 2007). The ruler covered almost completely the horizontal extent of the visual display, ranging from 42° to the left to 42° to the right. Accordingly, each tick of the ruler (*n* = 85) was 1° apart. Two-digit, random numbers were presented alternating above and below each tick. This set the spatial resolution of our behavioral paradigm.

Based on the individual availability of our participants, we collected all data on one to 4 different days. The experiment was presented in 12 blocks with 21 trials in each block. During each block about the same number of TMS and noTMS trials should be delivered in order to keep the participants unaware about the upcoming trial being a TMS or noTMS trial. Having 50% of TMS and noTMS trials per block would have resulted in total in twice the number of noTMS compared with TMS trials since we stimulated 2 areas (hMST and the control site) interspersed with noTMS trials in both cases. In order to adjust for this different numbers of TMS and noTMS trials, we decided to TMS-stimulate in 12 of the 21 trials per block, resulting in 9 noTMS trials per block. In total, this gave us 57% of TMS trials throughout this study, half of them over hMST and the other half over the controlsite.

Participants had a several minute rest between blocks, depending on the individual tiredness level. In each block, we delivered a train of 3 TMS pulses during 12 (57%) of the trials, at pseudorandomized intervals. Each TMS train (10 Hz, evenly spaced at *dt* = 100, 200 ms duration) was initialized 100 ms before self-motion stimulus onset (Fig. 4*B*). In half of the blocks, TMS was performed over the right-hemisphere hMST [targetTMS (T)], and in the other half of the blocks it was delivered over the nearby control site (first control condition, C1). The different stimulation sites were presented in a different pseudorandomized order for each participant. In the remaining interleaved 43% of the trials (9 per block), the TMS-coil remained over the area that was being stimulated in the same block, but no pulse was delivered. The latter are referred to as noTMS controls (C2). Note that, since no participant showed a significant difference between noTMS data collected during hMST blocks versus C1 blocks, we combined all of the C2 data from all blocks in our analysis.

#### Experimental Task

During the trials, participants were asked to fixate the central target. Trials with eye movements or blinks were not included in our analysis.

In the third phase of each trial, that is, after the movement phase, participants had to report the number on the ruler stimulus that was closest to their perceived heading by using a regular keyboard. The numbers of the ruler stimulus were 1° apart and shown in different random order in each trial.

#### TMS Session Protocol

For our experiment, we used a Magstim Rapid^2^ 70 mm (Magstim, UK) figure-of-eight coil with air cooling [AirFilm Coil (AFC—Rapid Version)] for TMS stimulation. For both stimulation areas, the handle of the coil was oriented 45° to the sagittal plane with the handle pointing to the back of the head, based on the coil orientation of a previous study stimulation area hMT/V5+ (Sack et al. 2006). This position was kept as similar as possible between all participants. The motor threshold was identified for each participant before the experiment (mean for all 8 participants: 65% of the maximum stimulator output) by stimulating over the hand motor area. The resting motor threshold was defined as smallest output value that was able to induce visible finger twitch in 3 out of 6 stimulations (Rossini et al. 1994). In this experiment, we stimulated with 110% of each participant’s threshold. All of our stimulation parameters were within established safety limits (Rossi et al. 2009). Some participants reported slight discomfort during the TMS stimulation, perhaps due to proximity to neck nerves or muscles, but did not report any difference between the experimental blocks and the control site blocks. Throughout the experiment, the participants as well as the experimenter wore earplugs to dampen the discharging noise of the TMS coil.

#### Analysis

During analysis, response numbers (i.e., perceived self-motion direction along the ruler) were converted to visual angle based on their position on the monitor in order to represent perceived heading. Only trials in which participants typed in a number actually presented on the monitor were considered for further analysis (~99.7% of all trials). The online inspection of the eye position guaranteed fixation throughout the whole trial since trials with blinks or eye movements were aborted and repeated again.

For the following analysis steps, the responses of the participants were analyzed separately for the 3 different self-motion directions. Two different exclusion criteria were applied to remove responses based on typing errors. First, perceived headings with a different polarity than the presented heading (perceived heading to the right when self-motion to the left was presented and vice versa) occurred in approximately 1.2% of all trials. We assumed that these erroneous responses were given accidentally and hence removed them prior to further data analysis. Second, we calculated the mean over all perceived headings for each stimulation condition per participant and excluded responses differing more than 3 times the standard deviation from this mean (~3% of all trials).

The mean of the perceived headings for each stimulation condition and each presented self-motion direction was considered as each participant’s individual baseline. The absolute values of the differences of the single responses to the corresponding baseline were averaged per participant and displayed as variance of the responses.

We calculated 3 repeated-measures ANOVAs (RM-ANOVAs) to compare the data collected in the hMST stimulation condition in a first test with the data collected in the control condition 2 (noTMS stimulation) and in a second test we compared data collected in the control conditions 1 and 2. In the third test, we compared data collected in the 2 TMS stimulation conditions (hMST vs. control area). In order to test our hypothesis, data were further analyzed using paired, one- or two-tailed *t*-tests.

## Results

### fMRI Localizer Data

In order to be as precise as possible in delivering the TMS pulses over the human functional equivalent of macaque area MST (hMST; Huk et al. 2002), participants took part in a fMRI localizer task. We chose the TMS stimulation sites based on individual fMRI scans. Figure 1*B* shows the functional data of one representative participant. In color, we marked the areas that were activated by the self-motion stimulus (*P* < 0.05). Different colors indicate data from the 2 different presentation conditions, that is, visual motion only in the left (activation shown in blue) or right (activation shown in yellow) part of the visual field.

We identified area hMST in each participant as part of the human motion complex activated not only by contra-, but also by ipsi-lateral stimulation (Dukelow et al. 2001). The exact Talairach coordinates for all the participants can be found in Table 1. Values were in line with previous studies identifying human area MST (Dukelow et al. 2001; Cardin et al. 2012). Table 1 also reports the exact coordinates of each participant’s control area, which we stimulated in our first control condition (C1).

**Table 1:** Talairach coordinates of area hMST and the control area (for control condition C1) for each participant as identified by the localizer stimulus

S1	hMST (41, −69, 4)	Control (33, −79, 4)
S2	hMST (49, −62, −2)	Control (38, −73, −2)
S3	hMST (48, −60, 1)	Control (39, −71, 1)
S4	hMST (39, −63, 7)	Control (29, −80, 10)
S5	hMST (42, −61, 4)	Control (36, −78, 4)
S6	hMST (42, −67, −3)	Control (29, −82, −3)
S7	hMST (42, −67, 6)	Control (31, −85, 4)
S8	hMST (43, −56, −2)	Control (33, −76, −2)
Mean (SD)	hMST [43(3), −63(4), 2(4)]	Control [34(4), −78(5), 2(4)]

### Deriving Our Model-Based Hypothesis

**Figure 5:**
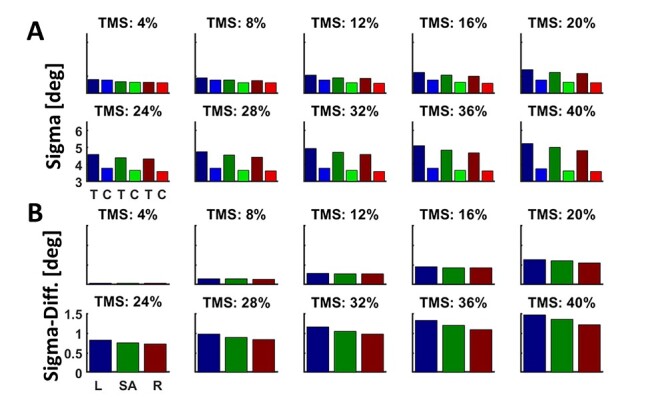
Effect of aTMS on heading decoding variance. Graphs in panel *A* depict the decoding variance for leftward (contraversive, blue), straight-ahead (green), and rightward (ipsiversive, red) self-motion. Each pair of bars indicates the result with (darker colors) and without (lighter colors) aTMS. We varied the strength of aTMS between ±4% and ±40% of the discharge of each neuron in steps of 4%. Graphs in panel *B* depict the difference in variance (with and without aTMS) for the 3 headings. For aTMS being ±20% or stronger, the variance in decoding for leftward (contraversive) self-motion was significantly stronger than for straight-ahead or rightward (ipsiversive) self-motion (ANOVA on ranks, *P* < 0.05, Bonferroni corrected for multiple comparisons). Likewise, for aTMS being 28% or stronger, decoding variance for straight-ahead self-motion was significantly stronger than for rightward self-motion (ANOVA on ranks, *P* < 0.05, Bonferroni corrected for multiple comparisons).

It was our goal to predict the outcome of our TMS stimulation based on a recently introduced model of heading representation (Bremmer et al. 2017). Importantly, this model was derived from neurophysiological recordings in the monkey. In our current work, we considered TMS as a disturbance of neural activity and in detail as inducing noise, that is, as inducing suppression and enhancement of neural activity in an equal proportion of neurons (Hallett 2007). In an extension of our model, we applied “artificial TMS” (aTMS) to a pool of model neurons. These neurons were built after response properties which we had reported previously (see Materials and Methods for details) (Bremmer et al. 2010; Greenlee et al. 2016). Briefly, we created a sample of *n* = 2000 artificial neurons, of which 45% (*n*_contra_ = 900) were tuned for contraversive (leftward) heading, 35% (*n*_ipsi_ = 700) for ipsiversive (rightward) heading and 20% (*n*_straight-ahead_) = 400 for straight-ahead self-motion (Fig. 2). We added noise to the activity of a given neuron to mimic real discharges as closely as possible and then applied aTMS to these neurons. In a final step, we decoded heading from a population of neurons and determined decoding variance with and without aTMS (Fig. 5). We repeated this procedure 100 times. As can be easily seen, mean response variance increased with increasing TMS strength. We tested the aTMS effect for significant differences for the 3 heading directions. For an aTMS value of 20% and larger, the aTMS-induced increase of variance was significantly larger for leftward (i.e., contraversive) self-motion as compared with straight-ahead and rightward self-motion (ANOVA on ranks, *P* < 0.05, Bonferroni-corrected for multiple comparisons). For an aTMS value of 28% and larger, also the difference of the variances for straight-ahead and rightward self-motion became statistically significant (*P* < 0.05, Bonferroni-corrected for multiple comparisons). Our experimental approach did not allow testing for the dynamics of TMS stimulation. Instead, the 3 TMS pulses were centered on self-motion onset to maximize the effect of stimulation. Accordingly, and based on our model prediction, for our behavioral experiment, we hypothesized disturbance of heading perception to be strongest for heading contraversive to the stimulated hemisphere, as indicated by a greater variance of the subjects’ heading perception. Our hypothesis was to see the above described disturbed heading perception after stimulation of area hMST with TMS pulses, but not when the control area was stimulated.

### Behavioral and TMS Data

In the behavioral/TMS part of our study, we investigated influence of TMS on the perceived heading of our participants. In a given trial, subjects were presented optic flow stimuli simulating self-motion across a ground plane in one of 3 directions (30° to the left, straight ahead or 30° to the right). At the end of each trial participants were presented a ruler stimulus and had to indicate the number which represented their self-motion direction via keyboard input.

**Figure 6:**
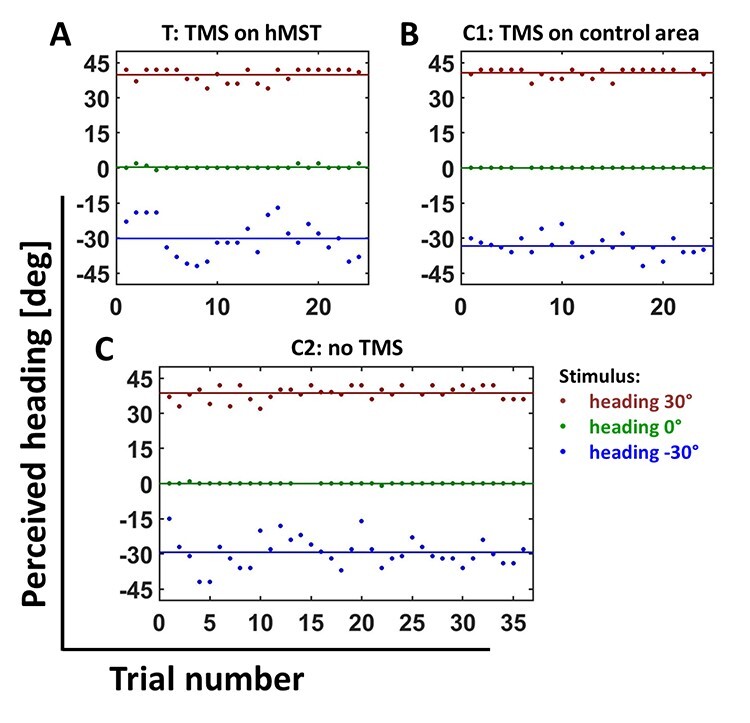
Perceived headings of one participant. In red data recorded in trials with a self-motion stimulus 30° to the right are presented, in green for self-motion straight forward (0°), and in blue for self-motion 30° to the left. The perceived headings are plotted in degrees with headings to the left with negative values and 0° as straight forward. We show the response data of one participant, here in the order it was collected. Each dot represents the response in a given trial. The mean of all these single trial responses is plotted as a horizontal line for each direction and condition. *A* shows the data recorded after stimulation of area hMST [targetTMS (T)], *B* shows the data recorded in control condition 1 (stimulation of the control area), and *C* shows the data of control condition 2 (noTMS stimulation).

Figure 6 shows the trial-by-trial perceived heading data for a single participant. The 3 panels depict results from the 3 different stimulation conditions: (1) TMS over hMST [targetTMS (T)], (2) first control condition (C1): TMS over the control area, and (3) second control condition (C2): noTMS. In the panels, each dot depicts the percept of the participant in a given trial. We calculated the mean of all responses separately for the 3 different self-motion directions and plotted theses values as colored solid lines (data for rightward self-motion in red, for straight ahead self-motion in green, and for leftward self-motion in blue). Note that this particular participant overestimated heading to the right (but not the other directions) by about 12°, irrespective of the stimulation condition, but this was not a consistent trend across subjects.

#### Statistical Analysis: Single Participant

As can be seen in the data of a single participant plotted in Figure 6, the single trial responses varied the most from the calculated mean in the “TMS over hMST” condition (mean variance of responses = 3.25°). Further, this condition showed the largest variance for self-motion to the left (variance of responses left = 6.57°), less so for self-motion to the right (variance of responses right = 2.56°) and almost no variance for self-motion straight forward (variance of responses straight ahead = 0.61°). Importantly, and in line with our model-based hypothesis, TMS over hMST did not shift the overall perception of heading. In other words, regardless of the simulated self-motion direction, average values of the responses for a given self-motion direction in the TMS condition were not significantly different from those in the 2 control conditions (participant 05: ANOVA with the responses of the participant; no significant main effect for stimulation condition: *F*(2.0, 238) = 0.23, *P* > 0.05).

**Figure 7:**
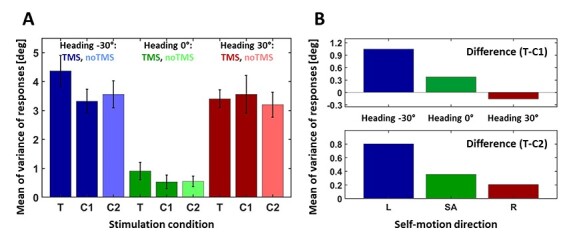
Mean variance and difference between T and the 2 controls (C1 and C2) for all participants. *A* shows the mean of variances with the standard errors of all 8 participants’ responses for the different stimulation conditions. For each self-motion direction, the 3 stimulation conditions (TMS over area MST [targetTMS (T)], control condition 1 (C1: stimulation of control site), and control condition 2 (C2: noTMS) are presented. In blue data of the self-motion direction to the left is shown, with light blue for noTMS trials. In green the self-motion direction straight forward is plotted, with light green for noTMS trials. In red the self-motion direction to the right is presented, with light red for noTMS trials. In the upper panel of *B*, we present the difference between condition T, stimulation of area MST, and condition C1, stimulation of the control area. In the lower panel, we depict the difference between T and condition C2, noTMS stimulation. As in *A*, data are shown for the 3 heading directions, to the left in blue, straight ahead in green, and to the right in red. Note that the differences for SA and R are not statistically significant.

As in Bremmer et al. (2017), overall accuracy of heading perception varied across subjects and, as shown in the example above, sometimes even within subjects for the different heading directions. Accordingly, for our population analysis, we normalized data per subject and heading direction. To this end, we subtracted for each trial per subject and heading direction the mean perceived heading. Figure 7*A* shows the variance of the heading perception across all subjects and heading directions for the 3 different experimental conditions, that is, (1) TMS over hMST [targetTMS (T)], (2) TMS over the control area (control condition 1, C1), and (3) noTMS (control condition 2, C2). Darker colors indicate data from stimulation trials (hTMS and C1), while lighter colors indicate data from trials without TMS (C2). Consistent with the single participant data in Figure 6, the variance for simulated self-motion directed straight ahead showed the smallest values. The variances for the self-motion direction to the right and to the left had larger values. Most importantly, the control data (C1 and C2) showed very similar patterns, whereas TMS over hMST produced higher variance, and most strongly for leftward (contraversive) headings.

#### Statistical Analysis: All Participants

To formally test these observations, we calculated 3 repeated-measures ANOVAs (RM-ANOVAs) before we calculated *t*-tests to evaluate our hypothesis in more detail. In the first RM-ANOVA, we contrasted data from the targetTMS condition and data from control condition 2. In the second RM-ANOVA, we compared the 2 control conditions C1 and C2. In addition to those 2 tests, we calculated an RM-ANOVA to compare data from the targetTMS condition (T) with data after TMS stimulation of the control area, the first control condition (C1).

According to our hypothesis about the importance of area hMST for self-motion perception, we expected to find a difference between data of TMS trials (T) and noTMS trials (C2), especially for leftward heading. In contrast, the control area (condition C1) was chosen to be not involved in the processing of self-motion information and therefore data collected in those trials (C1) should not be different from data collected in noTMS trials (C2). In addition, we hypothesized to see a difference between the 2 TMS stimulation sites, that is, a greater response variance for stimulation of hMST (condition T) compared with stimulation of the control site (condition C1), with the strongest effect for leftward heading.

In all 3 RM-ANOVAs, we found a significant main effect of heading direction (T vs. C2: *F*(1, 7) = 20.96, *P* < 0.01, effect size *f*: *f* = 1.73; C1 vs. C2: *F*(1, 7) = 15.82, *P* < 0.01, effect size *f*: *f* = 1.5; T vs. C1: *F*(1, 7) = 20.48, *P* < 0.01, effect size *f*: *f* = 1.71), which obviously resulted from the large differences in variance for heading straight ahead compared with heading to the left or right.

#### Comparison of T Versus C2 Trial Data

For the comparison of T versus C2 trial data, our RM-ANOVA revealed a significant main effect of stimulation condition (*F*(1, 7) = 6.2, *P* < 0.05, effect size *f*: *f* = 0.94). Hence, we conclude that distraction created by feeling the TMS stimulus could not explain our experimental findings. In order to test our hypothesis that TMS pulses over area hMST show an enhanced variance most strongly for heading to the left, we calculated paired, one-tailed *t*-tests to compare conditions T and C2 for all 3 self-motion directions (heading: left: *t*(7) = 2.78, *P* < 0.05; heading: straight ahead: *t*(7) = 1.51, *P* > 0.05; heading: right: *t*(7) = 0.47, *P* > 0.05). Further, Cohen’s effect size value (left: *d* = 0.56; straight ahead: *d* = 0.51; right: *d* = 0.19) suggested medium (for the self-motion directions left and straight ahead) to low (for the self-motion direction right) practical significance. Accordingly, after a Bonferroni correction for multiple comparisons, we found a significantly higher variance in responses for trials with TMS stimulation over hMST compared with noTMS data (C2) only for heading to the left, as shown in Figure 7*A*. The higher value of condition T compared with the value of control conditions 2 for self-motion straight ahead and to the right, which can be seen in Figure 7*B* as well, did not show a significant effect. In Figure 7*B*, we plotted the difference between the TMS condition with stimulation of MST and the control condition without TMS stimulation. Similar to the model data in Figure 5*B*, the differences calculated with the recorded data (Fig. 7*B*, lower panel) show an increase of variance for all 3 heading directions after TMS stimulation with the highest value for self-motion to the left. Yet, this increase of variance for self-motion to the right (ipsiversive) could only be observed for the comparison of T and C2 but not C1 (see upper and lower panels of Fig. 7*B*). Accordingly, while our model suggested a graded effect also for ipsiversive heading, depending on the effective aTMS strength, our experimental data do not allow to unequivocally draw this conclusion.

#### Comparison of C1 Versus C2 Trial Data

In a second analysis step, we wanted to further test for the influence of the control area (C1) on heading perception and compared data recorded after TMS stimulation of the control site (C1) with data recorded after noTMS trials (C2). We calculated paired, two-tailed *t*-tests for all 3 self-motion directions (heading: left: *t*(7) = −0.52, *P* > 0.05; heading: straight ahead: *t*(7) = −0.21, *P* > 0.05; heading: right: *t*(7) = 1.02, *P* > 0.05). As expected, TMS stimulation of the control area did not show a significant influence on heading perception compared with noTMS stimulation trials.

#### Comparison of T Versus C1 Trial Data

In a last analysis step, we wanted to test our hypothesis that only data after TMS pulses over area hMST show an enhanced variance, but not data after TMS stimulation of the control site. We calculated paired, one-tailed *t*-tests to compare conditions T and C1 for all 3 self-motion directions (heading: left: *t*(7) = 2.72, *P* < 0.05; heading: straight ahead: *t*(7) = 1.54, *P* > 0.05; heading: right: *t*(7) = −0.23, *P* > 0.05). Further, Cohen’s effect size value (left: *d* = 0.77; straight ahead: *d* = 0.49; right: *d* = 0.11) suggested high (for the self-motion directions left) to medium (for the self-motion directions straight ahead and right) practical significance. After a Bonferroni correction for multiple comparisons, we found a significantly higher variance in responses for trials with TMS stimulation over hMST compared with trials with TMS stimulation of the control site (C1) only for heading to the left. We did not find a significant difference between the 2 TMS stimulation areas for the other 2 heading directions.

Overall the results presented here, support our hypothesis: We found a higher variance in responses for self-motion when right hMST was stimulated, relative to both the control site and noTMS controls. Effects were strongest for contraversive heading. In contrast, stimulation over the control site produced no significant effect.

## Discussion

A number of previous fMRI studies in humans have shown strong correlations between visually simulated self-motion stimuli and neural activation in the functional equivalent of macaque medial superior temporal area MST, that is, area hMST (e.g., Morrone et al. 2000; Wall and Smith 2008; Pitzalis et al. 2013; Strong et al. 2017a). In monkeys, microstimulation experiments and studies employing reversible inactivation have provided clear evidence for a causal involvement of area MST in heading perception (stimulation: Britten and van Wezel 1998; inactivation: Gu et al. 2012). To our best knowledge, such proof of a causal involvement of area hMST in heading perception is lacking as of today. In a neuro-navigated approach, we applied TMS to area hMST and a control area slightly posterior while participants solved a heading perception task. Based on a recent study combining neurophysiological recordings in monkeys, modeling and a behavioral task in humans (Bremmer et al. 2017), we hypothesized that TMS over hMST, but not over the control site, should increase the variance of heading perception. As derived from our extended model, we predicted this decrease in precision to be strongest for self-motion directions contraversive with respect to the stimulated hemisphere (relative to noTMS controls). Results were in line with our hypothesis and thus provide the first evidence for a causal involvement of area hMST in heading perception in humans.

### Overall Performance

Our behavioral data revealed a general larger variance in heading perception for peripheral as compared to straight ahead self-motion. This is in line with previous studies showing that heading straight ahead is perceived more accurately than self-motion directions towards a peripheral direction (Crane 2012). The variances for peripheral headings were in the range of 3°–4° and, hence, in line with expectations based on previous findings (Warren and Hannon 1988). For many participants, the average perception for peripheral headings was closer to straight ahead than veridical (reduced accuracy). This was expected since the presentation time for our stimuli was only 50 ms. It had been shown before that accuracy in heading perception increases with presentation time for such ultra-short self-motion stimuli (Lich and Bremme 2014).

### Stimulation Effects in Visual Motion Processing

In humans, TMS is a noninvasive approach that can modulate neural processing and demonstrate any existing causal relationship between neural activity and behavior (Rossi et al. 2009; Prime et al. 2010; Thut and Pascual-Leone 2010; Vesia et al. 2010; Taylor and Thut 2012; Valero-Cabré et al. 2017). In previous studies, TMS had already been used to examine brain areas important for visual motion processing (e.g., Hotson and Anand 1999; Théoret et al. 2002; Sack et al. 2006; Laycock et al. 2007; Strong et al. 2017a, 2017b, 2019). As an example, TMS was applied over the V5/MT+ complex (Hotson and Anand 1999; Sack et al. 2006; Laycock et al. 2007) and was shown to impair visual motion processing: the proportion of visual elements moving coherently in the frontoparallel plane in a given direction had to be increased under TMS stimulation to obtain perceptual performance comparable with noTMS trials. Théoret et al. (2002) employed radial motion patterns, similar to those experienced during self-motion, to investigate the role of hMT for perceiving and storing the visual motion aftereffect (MAE). More specifically, the authors applied repetitive TMS (rTMS) during a storage interval and while subjects perceived the illusory motion. Indeed, rTMS over hMT disrupted the perception of the MAE when delivered early during the storage period and during the perceptual MAE itself. Also, Strong et al. (2017a) probed the perception of self-motion-like radial and rotational motion. They found that TMS over hMST, “but not hMT,” impaired visual motion processing. Further insight into the functional, causal role of areas hMT and hMST for visual motion perception was obtained from electrical stimulation experiments by Becker et al. (2013) and Campana et al. (2016). Becker and colleagues applied electrical stimulation over areas hMT and hMST in a patient undergoing awake brain surgery. Remarkably, the stimulation of both, areas hMT and hMST, made it impossible for the patient to perceive the global visual motion of moving random dot patterns. Campana and colleagues investigated the effects of transcranial random noise stimulation (tRNS) on motion adaptation and recovery. They found that, when applied bilaterally to hMT+, high-frequency tRNS caused a significant decrease in MAE duration, whereas low-frequency tRNS caused a significant increase. All these studies have already provided strong evidence for a causal role of areas hMT and hMST for visual motion processing. Importantly, among some of them the stimuli used were those typically occurring during self-motion, that is, radial expanding and contracting stimuli (Duffy and Wurtz 1991). Accordingly, our results, which show for the first time a causal involvement of area hMST for human heading perception, complement previous studies combining TMS and visual motion perception.

### Selective Effects of TMS on Heading Perception

**Figure 8:**
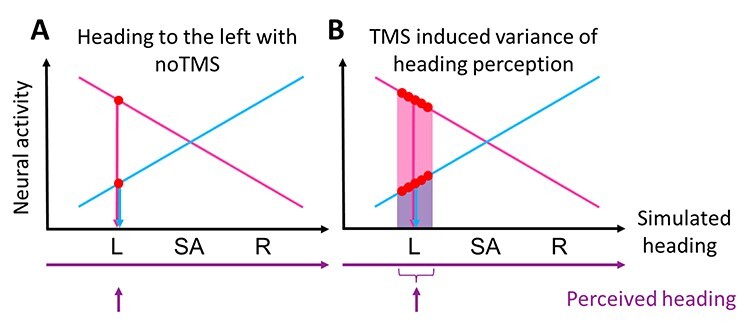
Model of the role of TMS on heading perception. Results are shown for self-motion to the left. MST neurons split up in two main subpopulations (magenta and blue), which show opposite tunings for the 3 heading directions left (L), straight ahead (SA) and right (R). The representation of a smaller subpopulation of neurons with strongest response for straight-ahead self-motion is not shown here. Without TMS stimulation, presented in *A*, the subpopulation coding for the presented heading (in this example: left) shows a high activation, while the subpopulation of neurons coding for the opposite direction (in this example: right) shows low activity. TMS stimulation was modeled as a disturbance of neural activity, that is, excitation and inhibition in a roughly equal number of neurons. This resulted in a greater variance of perceived heading as depicted in *B*.

Accuracy of heading-perception was not affected in our experiment, but precision. We consider this result, although predicted, as highly remarkable. Different from the above-mentioned findings of a detrimental effect of TMS on overall visual motion perception, accuracy was not affected by TMS. Our results do not allow, however, to judge if the sensitivity to self-motion stimuli was affected by TMS. While this is plausible, future studies will be necessary to investigate this issue. In any case, mean error, that is, the distance between average perceived and veridical heading, was not increased by TMS. This result was predicted by our model, as was an increased variance of perception, or a decrease of its inverse, that is, precision. Our model, as detailed in Bremmer et al. (2017) and shown schematically below (Fig. 8) assumes that the system has learned to associate a certain distribution of activities across a population of neurons in area hMST with a given heading. Let us assume we consider self-motion to the left. In the unperturbed state (Fig. 8*A*), this heading direction is associated with a relatively high activity of a subpopulation of neurons tuned for self-motion to the left and relative low activity in a subpopulation of neurons tuned for self-motion to the right. As shown earlier (e.g., Takahashi et al. 2007; Greenlee et al. 2016), there are comparably few neurons tuned for self-motion straight ahead. TMS is supposed to induce excitation and inhibition alike. Accordingly, we can assume that—across trials—increased and reduced activity, occur at equal proportion. In line with published literature, we further assume that perceptual readout is dominated by those neurons with the highest activity (Salzman and Newsome 1994). In our model, activity directly translates into heading direction. A TMS-induced higher activity of the neurons tuned for contraversive heading would shift the heading percept further into the periphery. Likewise, a TMS-induced reduction of activity would lead to a percept closer towards straight ahead. The opposite effect occurs for neurons tuned for rightward (ipsiversive) heading, while a mixed effect is supposed to occur for neurons tuned for straight-ahead self-motion. Accordingly, within and/or across trials, our qualitative model predicts an increase of perceptual variance with overall accuracy unaffected. This is what we found, and it provides further evidence for the idea that TMS introduces noise into the neural system, with excitation and inhibition alike (Hallett 2007; Valero-Cabré et al. 2017).

In some of our subjects, response variance for contraversive heading was slightly increased also in the 2 control conditions, as shown in the single subjects data (Fig. 6). While this effect was not as strong as in the test condition, that is, TMS over hMST, it is nevertheless noteworthy. While having no definitive answer to the question, why this was the case, we speculate that the effects of TMS stimulation outlasted the length of a given trial. Given that the effect was supposed to be strongest for contraversive heading, this assumption would predict (slightly) increased variance for all responses for contraversive heading, with the strongest effect seen for TMS over hMST. This is what is seen in this participant.

For our experiment, we invited 8 participants. While this number resulted from our power analysis performed prior to our experiments, it is admittedly a rather small number of participants. With this cohort, we found a significantly larger increase of TMS-induced variance of heading perception for contraversive as compared with straight-ahead and ipsiversive self-motion. Our model suggested that, depending on the effective strength of (a) TMS, a difference might also be observable between straight-ahead and ipsiversive self-motion. Future studies involving a larger group of participants combined with a parametrization of the effective TMS strength could allow to test for such finer grained differences. Along this line, variability of TMS results across previous studies even in other contexts than motion perception might have been due to different effective TMS strengths. Future studies involving a larger group of participants combined with a parametrization of the effective TMS strength could allow to test for such finer grained differences in self-motion perception specifically, and variability of TMS effects more generally.

### Timing of TMS

In our study, we stimulated always with 3 TMS pulses, separated by 100 ms and centered on self-motion onset, to maximize the influence of the stimulation. This setting was mainly based on a previous TMS study stimulating area hMT/V5+ (Sack et al. 2006). Here, Sack and colleagues documented an impairment in visual motion detection for TMS pulses presented in time windows of 40–30 ms before and 130–150 ms after visual motion onset. Since we did not have a clear hypothesis about what the optimal timing for a TMS pulse would be we applied 3 pulses as mentioned above, thereby roughly covering the interval tested by Sack and colleagues. It would be interesting to investigate in future experiments the dynamics of heading perception by varying the timing between TMS pulses and visual motion onset.

### TMS Stimulation Site

In order to probe the role of hMST for heading perception, a first and crucial step was to localize the area at an individual subject level to be able to direct our TMS stimulation at this target area. We used a functional localizer (as in Dukelow et al. 2001; Smith et al. 2006) and collected fMRI data from each of our participants. Talairach coordinates were well in line with published data (Dukelow et al. 2001; Cardin et al. 2012). The results provided by our localizer stimulus showed the expected activations for moving stimuli compared with stationary stimuli contralateral with the stimulation site. In addition, for each participant, we found areas with contra- and ipsi-lateral activation which we considered to be area hMST according to previous studies (Dukelow et al. 2001; Smith et al. 2006).

We decided to use a control area which was only about 1.5 cm posterior to area hMST, similar to the study by Dessing et al. (2013). Importantly, we made sure that this area did not show any difference in fMRI activation when comparing the BOLD response for presenting moving versus stationary dots. We used neuronavigated TMS in order to stimulate hMST and the control area. Since area hMT and hMST are located next to each other (Fig. 1), it might still have been the case that we also stimulated parts of area hMT. For the objective of our study, that is, the disturbance of heading perception, this, however, should not have been a problem because area hMST is known to be more important for the perception of self-motion direction than area hMT (Cardin et al. 2012).

## Conclusion

Here, we used neuro-navigated TMS to modulate neural processing in human MST during concurrent perception of visually simulated self-motion across a ground plane. As predicted, the variance in heading perception increased most strongly for heading contraversive to the stimulated hemisphere. This result provides further strong evidence for a causal involvement of hMST in the processing of self-motion information.

## Notes

*Conflict of Interest:* None declared.

## Funding

This work was supported by Deutsche Forschungsgemeinschaft (IRTG-1901, CRC/TRR-135 (project number: 222641018), Natural Science and Engineering Council of Canada (NSERC).

## References

[ref1] Angelaki DE , Gu Y, DeAngelis GC. 2011. Visual and vestibular cue integration for heading perception in extrastriate visual cortex. J Physiol. 589(4):825–833. 20679353 10.1113/jphysiol.2010.194720 PMC3060362

[ref2] Amano K , Wandell BA, Dumoulin SO. 2009. Visual field maps, population receptive field sizes, and visual field coverage in the human MT+ complex. J Neurophysiol. 102(5):2704–2718. 19587323 10.1152/jn.00102.2009 PMC2777836

[ref3] Becker HGT , Haarmeier T, Tatagiba M, Gharabaghi A. 2013. Electrical stimulation of the human homolog of the medial superior temporal area induces visual motion blindness. J Neurosci. 33(46):18288–18297. 24227738 10.1523/JNEUROSCI.0556-13.2013 PMC6619749

[ref4] Ben Hamed S , Page W, Duffy C, Pouget A. 2003. MSTd neuronal basis functions for the population encoding of heading direction. J Neurophysiol. 90(2):549–558. 12750416 10.1152/jn.00639.2002

[ref8] Bremmer F , Churan J, Lappe M. 2017. Heading representations in primates are compressed by saccades. Nat Commun. 8(1):920. 29030557 10.1038/s41467-017-01021-5 PMC5640607

[ref6] Bremmer F , Klam F, Duhamel J-R, Hamed SB, Graf W. 2002. Visual-vestibular interactive responses in the macaque ventral intraparietal area (VIP). Eur J Neurosci. 16:1569–1586. 12405971 10.1046/j.1460-9568.2002.02206.x

[ref7] Bremmer F , Kubischik M, Pekel M, Hoffmann K-P, Lappe M. 2010. Visual selectivity for heading in monkey area MST. Exp Brain Res. 200:51–60. 19727690 10.1007/s00221-009-1990-3

[ref5] Bremmer F , Schlack A, Shah NJ, Zafiris O, Kubischik M, Hoffmann K-P, Zilles K, Fink GR. 2001. Polymodal motion processing in posterior parietal and premotor cortex: a human fMRI study strongly implies equivalencies between humans and monkeys. Neuron. 29(1):287–296. 11182099 10.1016/s0896-6273(01)00198-2

[ref9] Britten KH , van Wezel RJA. 1998. Electrical microstimulation of cortical area MST biases heading perception in monkeys. Nat Neurosci. 1(1):59–63. 10195110 10.1038/259

[ref11] Britten KH . 2008. Mechanisms of self-motion perception. Annu Rev Neurosci. 31:389–410. 18558861 10.1146/annurev.neuro.29.051605.112953

[ref10] Britten KH , van Wezel RJA. 2002. Area MST and heading perception in macaque monkeys. Cereb Cortex. 12(7):692–701. 12050081 10.1093/cercor/12.7.692

[ref12] Campana G , Camilleri R, Moret B, Ghin F, Pavan A. 2016. Opposite effects of high- and low-frequency transcranial random noise stimulation probed with visual motion adaptation. Sci Rep. 6:38919. 27934947 10.1038/srep38919 PMC5146960

[ref13] Cardin V , Hemsworth L, Smith AT. 2012. Adaptation to heading direction dissociates the roles of human MST and V6 in the processing of optic flow. J Neurophysiol. 108:794–801. 22592304 10.1152/jn.00002.2012 PMC3424094

[ref14] Chen A , DeAngelis GC, Angelaki DE. 2011. Representation of vestibular and visual cues to self-motion in ventral intraparietal cortex. Journal Neurosci. 31(33):12036–12052. 10.1523/JNEUROSCI.0395-11.2011 PMC3169295 21849564

[ref16] Chen X , DeAngelis GC, Angelaki DE. 2018. Flexible egocentric and allocentric representations of heading signals in parietal cortex. PNAS. 115(14):E3305–E3312. 29555744 10.1073/pnas.1715625115 PMC5889634

[ref15] Chen A , Gu Y, Liu S, DeAngelis GC, Angelaki DE. 2016. Evidence for a causal contribution of macaque vestibular, but not intraparietal, cortex to heading perception. J Neurosci. 36(13):3789–3798. 27030763 10.1523/JNEUROSCI.2485-15.2016 PMC4812135

[ref17] Churan J , Paul J, Klingenhoefer S, Bremmer F. 2017. Integration of visual and tactile information in reproduction of traveled distance. J Neurophysiol. 118(3):1650–1663. 28659463 10.1152/jn.00342.2017 PMC5577551

[ref18] Crane BT . 2012. Direction specific biases in human visual and vestibular heading perception. PLoS One. 7(12):e51383. 23236490 10.1371/journal.pone.0051383 PMC3517556

[ref19] Dessing JC , Vesia M, Crawford JD. 2013. The role of areas MT+/V5 and SPOC in spatial and temporal control of manual interception: an rTMS study. Front Behav Neurosci. 7(15):1–13. 10.3389/fnbeh.2013.00015 PMC3587841 23468002

[ref20] Duffy C , Wurtz RH. 1991. Sensitivity of MST neurons to optic flow stimuli. I. A continuum of response selectivity to large-field stimuli. J Neurophysiol. 65(6):1329–1345. 10.1152/jn.1991.65.6.1329 1875243

[ref21] Dukelow SP , DeSouza JFX, Culham JC, van den Berg AV, Menon RS, Vilis T. 2001. Distinguishing subregions of the human MT+ complex using visual fields and pursuit eye movements. J Neurophysiol. 86(4):1991–2000. 11600656 10.1152/jn.2001.86.4.1991

[ref22] Faul F , Erdfelder E, Buchner A, Lang A-G. 2009. Statistical power analyses using G*Power 3.1: tests for correlation and regression analyses. Behavior Research Methods. 41:1149–1160. 19897823 10.3758/BRM.41.4.1149

[ref23] Gibson JJ . 1950. The perception of the visual world. Cambridge (MA): The Riverside Press.

[ref24] Greenlee MW , Frank SM, Kaliuzhna M, Blanke O, Bremmer F, Churan J, Cuturi LF, MacNeilage PR, Smith AT. 2016. Multisensory integration in self-motion perception. Multisens Res. 29:525–556.

[ref26] Gu Y , DeAngelis GC, Angelaki DE. 2012. Causal links between dorsal medial superior temporal area neurons and multisensory heading perception. J Neurosci. 32(7):2299–313. 22396405 10.1523/JNEUROSCI.5154-11.2012 PMC3311305

[ref25] Gu Y , Watkins PV, Angelaki DE, DeAngelis GC. 2006. Visual and nonvisual contributions to three-dimensional heading selectivity in the medial superior temporal area. J Neurosci. 26(1):73–85. 16399674 10.1523/JNEUROSCI.2356-05.2006 PMC1538979

[ref27] Hallett M . 2007. Transcranial magnetic stimulation: a primer. Neuron. 55:187–199. 17640522 10.1016/j.neuron.2007.06.026

[ref28] Heuer A , Schubö A, Crawford JD. 2016. Different cortical mechanisms for spatial vs. feature-based attentional selection in visual working memory. Front Hum Neurosci. 10:415. 27582701 10.3389/fnhum.2016.00415 PMC4987349

[ref29] Hlavacka F , Mergner T, Bolha B. 1996. Human self-motion perception during translatory vestibular and proprioceptive stimulation. Neurosci Lett. 210:83–86. 8783278 10.1016/0304-3940(96)12667-7

[ref30] Hotson JR , Anand S. 1999. The selectivity and timing of motion processing in human temporo-parieto-occipital and occipital cortex: a transcranial magnetic stimulation study. Neuropsychologia. 37:169–179. 10080374 10.1016/s0028-3932(98)00091-8

[ref31] Huk AC , Dougherty RF, Heeger DJ. 2002. Retinotopy and functional subdivision of human areas MT and MST. J Neurosci. 22(16):7195–7205. 12177214 10.1523/JNEUROSCI.22-16-07195.2002 PMC6757870

[ref32] Kaminiarz A , Krekelberg B, Bremmer F. 2007. Localization of visual targets during optokinetic eye movements. Vision Res. 47:869–878. 17178144 10.1016/j.visres.2006.10.015

[ref33] Kaminiarz A , Schlack A, Hoffmann K-P, Bremmer F. 2014. Visual selectivity for heading in the macaque ventral intraparietal area. J Neurophysiol. 112:2470–2480. 25122709 10.1152/jn.00410.2014

[ref34] Lappe M , Bremmer F, Pekel M, Thiele A, Hoffmann K-P. 1996. Optic flow processing in monkey STS: a theoretical and experimental approach. J Neurosci. 16(19):6265–6285. 8815907 10.1523/JNEUROSCI.16-19-06265.1996 PMC6579186

[ref35] Lappe M , Bremmer F, van den Berg AV. 1999. Perception of self-motion from visual flow. Trends Cogn Sci. 3(9):329–336. 10.1016/s1364-6613(99)01364-9 10461195

[ref36] Laycock R , Crewther DP, Fitzgerald PB, Crewther SG. 2007. Evidence for fast signals and later processing in human V1/V2 and V5/MT+: a TMS study of motion perception. J Neurophysiol. 98:1253–1262. 17634339 10.1152/jn.00416.2007

[ref37] Li M , Xie K, Kuang H, Liu J, Wang D, Fox GE, Wei W, Li X, Li Y, Zhao F, et al. 2018. Spike-timing pattern operates as gamma-distribution across cell types, regions and animal species and is essential for naturally-occurring cognitive states. bioRxiv 145813.

[ref38] Lich M , Bremme F. 2014. Self-motion perception in the elderly. Front Hum Neurosci. 8:681. 25309379 10.3389/fnhum.2014.00681 PMC4163979

[ref39] Morrone MC , Tosetti M, Montanaro D, Fiorentini A, Cioni G, Burr DC. 2000. A cortical area that responds specifically to optic flow, revealed by fMRI. Nat Neurosci. 3(12):1322–1328. 11100154 10.1038/81860

[ref40] Paolini M , Distler C, Bremmer F, Lappe M, Hoffmann K-P. 2000. Response to continuously changing optic flow in area MST. J Neurophysiol. 84(2):730–743. 10938300 10.1152/jn.2000.84.2.730

[ref41] Pitzalis S , Bozzacchi C, Bultrini A, Fattori P, Galletti C, Di Russo F. 2013. Parallel motion signals to the medial and lateral motion areas V6 and MT+. Neuroimage. 15, 67:89–100. 10.1016/j.neuroimage.2012.11.022 23186916

[ref42] Prime SL , Vesia M, Crawford JD. 2010. TMS over human frontal eye fields disrupts trans-saccadig memory of multiple objects. Cereb Cortex. 20:759–772. 19641017 10.1093/cercor/bhp148

[ref43] Romero MC , Davare M, Armendariz M, Janssen P. 2019. Neural effects of transcranial magnetic stimulation at the single-cell level. Nat Commun. 10(1):2642. 31201331 10.1038/s41467-019-10638-7 PMC6572776

[ref44] Rossi S , Hallett M, Rossini PM, Pascual-Leone A. 2009. The safety of TMS consensus group. Safety, ethical considerations, and application guidelines for the use of transcranial magnetic stimulation in clinical practice and research. Clin Neurophysiol. 120:2008–2039. 19833552 10.1016/j.clinph.2009.08.016 PMC3260536

[ref45] Rossini PM , Baker AT, Berardelli A, Caramia MD, Caruso G, Cracco RQ, Dimitrijevid MR, Hallett M, Katayama Y, Lücking CH, et al. 1994. Non-invasive electrical and magnetic stimulation of the brain, spinal cord and roots: basic principles and procedures for routine clinical application. Report of an IFCN committee. Electroencephalogr Clin Neurophysiol. 91:79–92. 7519144 10.1016/0013-4694(94)90029-9

[ref46] Saito H , Yukie M, Tanaka K, Hikosaka K, Fukada Y, Iwai E. 1986. Integration of direction signals of image motion in the superior temporal sulcus of the macaque monkey. J Neurosci. 6(1):145–157. 3944616 10.1523/JNEUROSCI.06-01-00145.1986 PMC6568620

[ref47] Sack AT , Kohler A, Linden DEJ, Goebel R, Muckli L. 2006. The temporal characteristics of motion processing in hMT/V5+: combining fMRI and neuronavigated TMS. NeuroImage. 29:1326–35. 16185899 10.1016/j.neuroimage.2005.08.027

[ref48] Salzman CD , Newsome WT. 1994. Neural mechanisms for forming a perceptual decision. Science. 264(5156):231–237. 8146653 10.1126/science.8146653

[ref49] Schlack A , Hoffmann K-P, Bremmer F. 2002. Interaction of linear vestibular and visual stimulation in the macaque ventral intraparietal area (VIP). Eur J Neurosci. 16:1877–1886. 12453051 10.1046/j.1460-9568.2002.02251.x

[ref50] Shao M , DeAngelis GC, Angelaki DE, Chen A. 2018. Clustering of heading selectivity and perception-related activity in the ventral intraparietal area. J Neurophysiol. 119:1113–1126. 29187554 10.1152/jn.00556.2017 PMC5899310

[ref51] Smith AT , Wall MB, Williams AL, Singh KD. 2006. Sensitivity to optic flow in human cortical areas MT and MST. Eur J Neurosci. 23(2):561–569. 16420463 10.1111/j.1460-9568.2005.04526.x

[ref52] Strong SL , Silson EH, Gouws AD, Morland AB, McKeefry DJ. 2017a. A direct demonstration of functional differences between subdivisions of human V5/MT+. Cereb Cortex. 27:1–10. 28365777 10.1093/cercor/bhw362 PMC5939194

[ref53] Strong SL , Silson EH, Gouws AD, Morland AB, McKeefry DJ. 2017b. Differential processing of the direction and focus of expansion of optic flow stimuli in areas MST and V3A of the human visual cortex. J Neurophysiol. 117:2209–2217. 28298300 10.1152/jn.00031.2017 PMC5454473

[ref54] Strong SL , Silson EH, Gouws AD, Morland AB, McKeefry DJ. 2019. An enhanced role for right hV5/MT+ in the analysis of motion in the contra- and ipsi-lateral visual hemi-fields. Behav Brain Res. 372:112060. 31251957 10.1016/j.bbr.2019.112060 PMC6682608

[ref55] Takahashi K , Gu Y, May PJ, Newlands SD, DeAngelis GC, Angelaki DE. 2007. Multimodal coding of three-dimensional rotation and translation in area MSTd: comparison of visual and vestibular selectivity. J Neurosci. 27(36):9742–9756. 17804635 10.1523/JNEUROSCI.0817-07.2007 PMC2587312

[ref56] Taylor PC , Thut G. 2012. Brain activity underlying visual perception and attention as inferred from TMS-EEG: a review. Brain Stimul. 5(2):124–129. 22494831 10.1016/j.brs.2012.03.003

[ref57] Théoret H , Kobayashi M, Ganis G, Di Capua P, Pascual-Leone A. 2002. Repetitive transcranial magnetic stimulation of human area MT/V5 disrupts perception and storage of the motion aftereffect. Neuropsychologia. 40(13):2280–2287. 12417458 10.1016/s0028-3932(02)00112-4

[ref58] Thut G , Pascual-Leone A. 2010. A review of combined TMS-EEG studies to characterize lasting effects of repetitive TMS and assess their usefulness in cognitive and clinical neuroscience. Brain Topogr. 22(4):219–232. 19862614 10.1007/s10548-009-0115-4 PMC3260526

[ref59] Valero-Cabré A , Amengual JL, Stengel C, Pascual-Leone A, Coubard OA. 2017. Transcranial magnetic stimulation in basic and clinical neuroscience: a comprehensive review of fundamental principles and novel insights. Neurosci Biobehav Rev. 83:381–404. 29032089 10.1016/j.neubiorev.2017.10.006

[ref60] Vesia M , Prime SL, Yan X, Sergio LE, Crawford JD. 2010. Specificity of human parietal saccade and reach regions during transcranial magnetic stimulation. J Neurosci. 30(39):13053–13065. 20881123 10.1523/JNEUROSCI.1644-10.2010 PMC6633525

[ref61] von Hopffgarten A , Bremmer F. 2011. Self-motion reproduction can be affected by associated auditory cues. Seeing Perceiving. 24(3):203–222. 21864463 10.1163/187847511X571005

[ref62] Wall MB , Smith AT. 2008. The representation of egomotion in the human brain. Curr Biol. 18:191–194. 18221876 10.1016/j.cub.2007.12.053

[ref63] Wall MB , Lingnau A, Ashida H, Smith AT. 2008. Selective visual responses to expansion and rotation in the human MT complex revealed by functional magnetic resonance imaging adaptation. Eur J Neurosci. 27:2747–2757. 18547254 10.1111/j.1460-9568.2008.06249.x

[ref64] Warren WH , Hannon DJ. 1988. Direction of self-motion is perceived from optical flow. Nature. 336:162–163.

[ref65] Zhang T , Britten KH. 2011. Parietal area VIP causally influences heading perception during pursuit eye movements. J Neurosci. 31(7):2569–2575. 21325524 10.1523/JNEUROSCI.5520-10.2011 PMC3084547

